# Cognitive profiles and associated structural brain networks in a multimorbid sample of marginalized adults

**DOI:** 10.1371/journal.pone.0218201

**Published:** 2019-06-13

**Authors:** Kristina M. Gicas, Andrea A. Jones, William J. Panenka, Chantelle Giesbrecht, Donna J. Lang, Fidel Vila-Rodriguez, Olga Leonova, Alasdair M. Barr, Ric M. Procyshyn, Wayne Su, Alexander Rauscher, A. Talia Vertinsky, Tari Buchanan, G. William MacEwan, Allen E. Thornton, William G. Honer

**Affiliations:** 1 Department of Psychiatry, University of British Columbia, Vancouver, BC Canada; 2 Department of Psychology, Simon Fraser University, Burnaby, BC Canada; 3 Department of Radiology, University of British Columbia, Vancouver, BC Canada; 4 Department of Anesthesiology, Pharmacology and Therapeutics, University of British Columbia, Vancouver, BC Canada; 5 Department of Paediatrics, University of British Columbia, Vancouver, BC Canada; Taipei Veterans General Hospital, TAIWAN

## Abstract

**Introduction:**

Cognition is impaired in homeless and vulnerably housed persons. Within this heterogeneous and multimorbid group, distinct profiles of cognitive dysfunction are evident. However, little is known about the underlying neurobiological substrates. Imaging structural covariance networks provides a novel investigative strategy to characterizing relationships between brain structure and function within these different cognitive subgroups.

**Method:**

Participants were 208 homeless and vulnerably housed persons. Cluster analysis was used to group individuals on the basis of similarities in cognitive functioning in the areas of attention, memory, and executive functioning. The principles of graph theory were applied to construct two brain networks for each cognitive group, using measures of cortical thickness and gyrification. Global and regional network properties were compared across networks for each of the three cognitive clusters.

**Results:**

Three cognitive groups were defined by: higher cognitive functioning across domains (Cluster 1); lower cognitive functioning with a decision-making strength (Cluster 3); and an intermediate group with a relative executive functioning weakness (Cluster 2). Between-group differences were observed for cortical thickness, but not gyrification networks. The lower functioning cognitive group exhibited higher segregation and reduced integration, higher centrality in select nodes, and less spatially compact modules compared with the two other groups.

**Conclusions:**

The cortical thickness network differences of Cluster 3 suggest that major disruptions in structural connectivity underlie cognitive dysfunction in a subgroup of people who have a high multimorbid illness burden and who are vulnerably housed or homeless. The origins, and possible plasticity of these structure-function relationships identified with network analysis warrant further study.

## Introduction

Excessive multimorbidity is a prominent feature of marginalized populations worldwide, including persons who are homeless or vulnerably housed [1]. Common co-occurring illnesses include psychosis, polysubstance use, HIV infection, and traumatic brain injury [2–4], which may reflect a set of genetic, environmental, and developmental vulnerabilities that predispose individuals to significant cognitive dysfunction. Previous studies in marginalized populations have shown substantial cognitive heterogeneity and impairment across domains [5–7] and this was linked with regional alterations in cortical thickness and gyrification [8], and to variation in white matter microstructure [9]. However, due to the scope of illness burden among marginalized persons, there are likely to be more widespread alterations to structural brain integrity, which warrants further exploration. Given that multimorbidity is an emergent global health issue [10], there is impetus for extending our knowledge of structure-function relationships by characterizing distinct neural and cognitive phenotypes that reflect the consequences of multiple co-occurring psychiatric and physical illnesses, as opposed to those associated with single diagnostic categories.

To better understand the neurobiological underpinnings of cognitive profiles in marginalized persons, brain network science using a graph theoretical approach provides an opportunity to move beyond traditional reductionist frameworks that focus on localized structure-function associations and consider cognition as an emergent property of distributed, interactive regions embedded within a complex system. Specifically, structural covariance networks enable us to examine how inter-individual differences in one anatomical region are correlated with inter-individual differences in another region. These patterns of brain covariation are presumed to reflect a product of coordinated anatomical development, maturation, or plasticity driven by both genetic and environmental factors and can be used to infer underlying structural connectivity and topological organization of the brain [11].

Few studies have explicitly linked structural brain networks with cognition. Diffusion tensor tractography studies in healthy adults have found that higher network efficiency and better integration of information are associated with higher overall intelligence [12,13]. Others have reported an association between network properties and intelligence only for adults aged 75 years and older [14]. In one of the most robust studies to date, Seidlitz and colleagues [15] used a novel structural covariance technique to construct brain networks that incorporated multiple indices of grey and white matter and found that network measures accounted for 40% of the overall variance in intelligence. Converging results from functional brain network analyses using resting state fMRI have reported a relationship between better integration and efficiency of brain networks and higher intelligence [16], while task-activated fMRI found a positive association between network efficiency and working memory only for younger healthy adults, with a negative association in older adults [17].

The current study used a structural covariance approach to examine brain network topologies underlying distinct cognitive profiles in a large cohort of marginalized persons recruited from a socially and economically impoverished community located in Vancouver, Canada. A network-based approach to evaluating patterns of cognitive functioning provides a meaningful way of understanding the neuroanatomical markers of dysfunction that more closely aligns with an integrated systems-based perspective of cognitive processing [18]. This study represents an important extension of our previous work that examined groups subtyped by cognitive strengths and weakness in the domains of premorbid intellectual functioning, attention, verbal memory, and executive functioning, and their association with regional variations in cortical architecture [6,8]. Three groups were described by profiles of higher functioning overall (Cluster 1), lower functioning overall with a relative strength in decision-making (Cluster 3), and a group that fell intermediate to the others but exhibited a prominent weakness in executive functioning (Cluster 2). These groups have also been previously well validated on the basis of clinical variables, expressing differential patterns of multimorbidity burden, including substance dependence, viral infection, and psychotic illness [6,8]. The clustering strategy therefore provides a complementary approach to using diagnostic categories by enabling us to capture co-variation in cognitive functioning that maximizes within-group similarities and between-group differences and, as a function of this, also captures distinct illness profiles that may be contributing to cognitive dysfunction.

This study aimed to describe and compare the network topology between distinct cognitive groups using surface-based measures of cortical thickness and gyrification. A secondary aim was to describe the modular composition within the network of each cognitive group. It was hypothesized that the structural networks of Clusters 2 and 3 would be characterized by poorer network efficiency and integration compared to Cluster 1 (highest functioning group). Further, it was anticipated that Cluster 1 would exhibit greater modularity and more spatially compact modules compared to the lower functioning Clusters 2 and 3.

## Materials and methods

### Participants

Data was used from the Hotel Study [3,4], an ongoing observational study of homeless and marginally housed persons living within an impoverished neighbourhood of Vancouver, Canada. Three hundred and seventy one (N = 371) participants were enrolled between November 2008 and November 2014. Participants were recruited from single-room occupancy (SRO) hotels (n = 306) and the community courthouse (n = 65) located within this neighbourhood. Enrollment eligibility included being 18 years of age or older, fluent in English, and providing written informed consent. Additionally, participants were required to be dwelling in one of four identified SRO hotels in the target neighbourhood or have had contact with the community court anytime within the previous six months. The sociodemographic and clinical characteristics of our sample are comparable to other studies of homeless and marginally housed persons in major urban centers across Canada [19,20]. The morbidities of our sample and their respective rates are also consistent with those reported in other developed nations [2]. There are no evident differences in health status or health care needs between those who are homeless and those who are vulnerably housed [19,21].

Written informed consent was obtained from all individual participants included in the study. To ensure participants had the capacity to provide full informed consent, they needed to be able to communicate fluently in English and indicate that they understood the nature and purpose of the study, the limits to confidentiality, and their right to withdraw at any time without consequence. They were provided with a minimum of 24 hours to consider the study information prior to being eligible for enrolment. If concerns emerged about capacity, a study psychiatrist was available for consultation (FVR, WGH). Consent was reaffirmed at each follow-up visit. Participants were provided with small cash honoraria for completion of each assessment. This study received ethics approvals from the Clinical Research Ethics Board of the University of British Columbia and the Simon Fraser University Office of Research Ethics.

### Cognitive and clinical assessments

A battery of cognitive tests was administered to participants by trained research assistants under the supervision of a registered psychologist (AET). The Wechsler Test of Adult Reading (WTAR) [22] was used to estimate premorbid full scale IQ (FSIQ). Verbal learning and memory was measured using the Hopkins Verbal Learning Test–Revised (HVLT-R) [23] total immediate recall score. The Cambridge Neuropsychological Test Automated Battery [24] was used to measure sustained attention (Rapid Visual Information Processing subtest [RVIP], A prime [A’] score) and mental flexibility (Intra-Dimensional Extra-Dimensional subtest [IDED], total adjusted errors score). The Stroop color-word subtest score was used to index cognitive inhibition. Finally, affective decision-making in the context of reward was measured using the Iowa Gambling Task total net score [25]. Examiners rated the validity of each cognitive test result on a scale from 1 (clearly invalid) to 5 (clearly valid). Data for tests rated as 3 (questionably valid) or lower were excluded from analyses. Reasons for invalid data may include participant intoxication, extreme fatigue, equipment malfunctions, or poor compliance with testing. English language fluency was assessed using the English Language Acculturation Questionnaire. Total scores range from 12 (very fluent in English) to 60 (not at all fluent in English), and those with a score of 24 (much fluent in English) or lower were included in analyses. These details of the cognitive assessment protocol have been previously published [6,8,9].

Clinical assessments were conducted at study entry by research psychiatrists. Diagnoses for psychiatric illness and substance dependence were made according to the Diagnostic and Statistical Manual of Mental Disorders 4^th^ edition [26] criteria and achieved by consensus using the Mini-International Neuropsychiatric Interview [27], the Best Estimate Clinical Evaluation and Diagnosis [28], and a mental status exam. The Charlson Comorbidity Index was used to measure co-occurring physical illnesses using the Charlson weighting scheme with one point added for every decade of life after 40 years [29]. A medical questionnaire was used to ascertain self-reported history of a traumatic brain injury. Blood tests were used to assess viral serology, where seropositivity indicated active infection for HIV and viral exposure for hepatitis C. Structured interviews with trained research assistants were conducted to collect demographic data.

### Neuroimaging acquisition and processing

Whole brain structural magnetic resonance imaging (MRI) scans were obtained at the time of, or proximal to, cognitive testing (99% within one month). Images were acquired on a 3.0T Phillips Achieva scanner with an 8-channel SENSE head coil. A 3D Fast Field Echo T1-weighted structural sequence was obtained in the sagittal plane with 190 1-mm thick slices (TR/TE = 7.6/3.5 ms; acquisition matrix = 256 X 250; field of view = 256 mm; flip angle = 8°; total acquisition time = 7:23 min).

FreeSurfer v5.1.0 (available at: https://surfer.nmr.mgh.harvard.edu/) was used to reconstruct the cortical surface using the standard processing stream. At each vertex of the reconstructed surface, the local gyrification index (lGI) was calculated as the ratio of the surface area within the sulci to the surface area that is exposed [30] and cortical thickness (CT) was calculated as the distance in millimetres between the pial surface and the gray-white matter boundary [31]. Visual inspections of the pial and white matter surfaces were conducted by trained raters (DJL, WS) and manual corrections were applied as required. This is the same procedure previously used with this data set [8]. Automatic cortical parcellation was based on sulcal and gyral divisions using the Destrieux atlas [32], yielding a total of 148 regions of interest (ROIs; 74 for each hemisphere). A standard Gaussian surface smoothing filter (10mm) was applied for cortical thickness ascertainment [33]. The lGI and CT values at each vertex were averaged separately to yield either a lGI or CT measure for each atlas-defined ROI.

### Cognitive cluster construction

A cluster analysis was used to group participants on the basis of common profiles of cognitive functioning. To summarize, a subsample of 299 participants had valid cognitive data and were missing no more than one test on the following set of variables: premorbid FSIQ, HVLT-R immediate recall, RVIP A’, Stroop color-word, IDED total adjusted errors, and IGT total net score. The IDED variable was log transformed due to a significant positive skew and multiplied by -1 to align it with interpretation of other cognitive measures. Next, cognitive scores were regressed on age and education, and the residual scores were submitted to a k-means cluster analysis specifying three groups. Groups were compared on demographic and clinical sample characteristics using ANOVAs (analysis of variance) and Kruskal-Wallis tests (non-parametric) for continuous variables and chi-square tests for categorical variables. This cluster analysis and group comparisons were previously performed on this subsample of 299 participants and were reported in detail elsewhere [8].

### Brain network construction

A subsample of 208 participants had available imaging data that were free from visible motion artifact for inclusion in the network analysis. This subsample remains nearly identical to our previous analyses limited to regional cortical measurements (n = 211) [8]. As a sensitivity analysis, participants who were included versus excluded from the network analysis were compared on demographic variables (age, sex, education, ethnicity, and estimated premorbid FSIQ) using t-tests and chi-square tests. Reasons for exclusion were not having completed a cognitive assessment or MRI scan; having more than one invalid or missing cognitive test; and/or poor scan quality (e.g., motion artifact, significant lesions resulting in segmentation failure.)

The procedure described below was used to independently construct gyrification- and cortical thickness-based brain networks using the Graph-Theoretical Analysis Toolbox implemented in MatLab [34]. As a first step, linear regression analysis was performed on each of the 148 ROIs to remove variance associated with age, sex, age by sex interaction, and either total gyrification or total cortical thickness. The regression residuals were then used to create a 148 X 148 Pearson correlation matrix for each of the three cognitive clusters. This approach has been recommended over using partial correlations when the sample size for a group is smaller than the number of ROIs [35]. Binarized adjacency matrices, which reflect unweighted and undirected graphs, were computed by setting all significant positive correlations (*p* < .05) to 1 and non-significant or negative correlations to 0. The diagonals of the matrices (correlation of a region with itself) were also set to 0. Only positive correlations were included because they are considered to more closely represent underlying fibre pathways, whereas negative correlations may reflect indirect connections and therefore have different biological meaning [36]. To control for noisy or spurious associations between regions, adjacency matrices were thresholded by density (the proportion of non-zero elements to all possible connections), which facilitates comparison of networks between groups. For cortical thickness networks, a minimum density of .07 (7%) was determined as the lowest value that rendered a fully connected (i.e., not fragmented) graph within each of the three groups, and increased by an interval of .39 to a maximum density of .46 (46%). For gyrification networks, the minimum density was .06 (6%), increasing by an interval of .38 to a maximum density of .44 (44%). Densities above 50% in structural networks approach a random configuration and are not considered to be biologically meaningful [37].

### Brain network properties

Binarized matrices were used to compute global and regional metrics for description and comparison of the network topologies across the three cognitive clusters. While there are many global metrics that can be computed to describe topological network properties, indices of segregation, integration, and small-worldness are of key interest [38–40]. It is prudent to seek convergence across metrics that measure similar network properties, thus we opted to examine two or more metrics, where appropriate, along each topological dimension. The following metrics that are described were computed with the GAT application in MatLab based on formulas provided by Rubinov and Sporns [40].

With respect to indices of network segregation, the *clustering coefficient* is one of the most common metrics used, and is defined as the average of the number of existing connections between the neighbors of a node divided by all possible connections. It is often considered to be a measure of network “cliquishness”, with higher values reflecting greater localized covariance. A related measure is *transitivity*, which is a normalized variant of the clustering coefficient and is less influenced by nodes with low degree. Transitivity is defined as the likelihood that two nodes are connected if they each have a connection to a common third node. Further, *local efficiency* was measured as the inverse of the shortest number of edges (i.e., correlations between regions) among neighbors of a given node. Higher local efficiency suggests better fault tolerance because it indexes how well a network transmits information following elimination of a given node.

Two complementary indices of network integration were also examined. First, we computed the *characteristic path length*, the most commonly reported measure of integration, defined as the average smallest number of edges required to connect two nodes. Shorter path length reflects stronger overall routing efficiency and is generally considered optimal because it minimizes conduction delays, susceptibility to noise, and energy requirements. We also calculated *global efficiency*, which is the inverse of the harmonic mean of the shortest path length between each node. Thus, a lower value for global efficiency is suggestive of weaker connectivity and poorer integration of information across modules.

Small-world architecture is taken to be a universal feature of complex networks and has been observed across a wide range of biological and non-biological systems [41]. *Small-worldness* is defined as the ratio of the normalized clustering coefficient to the normalized path length, where normalized values were derived by dividing a given coefficient by the same coefficient generated from 20 null networks (i.e., random graphs with the same number of nodes, edges, and degree distribution). As a simple rule-of-thumb, values greater than 1 were considered consistent with a small-world topology and can be interpreted as a measure of cost-efficiency whereby there is an ideal balance between local specialization and global integration.

On the other hand, regional metrics are also of interest because global measures lack specificity and may not have sufficient power to detect effects that are present at just a few nodes within the overall network. One important regional analysis includes the identification of hubs within the networks. Hubs represent nodes of relatively greater importance within a network because they have a high degree of connections with other nodes and therefore represent mediators of major information flow. Indices of *centrality* can be calculated to determine how influential a given node is within the network and therefore whether it is defined as a hub. A common measure of centrality is *node betweeness*, which is the fraction of all shortest paths that pass through a given node. A node was considered a hub if its node betweeness was at least two standard deviations higher than the mean network betweeness.

Modularity of each network was also evaluated. *Modularity* is an index of segregation and represents the division of nodes into non-overlapping subsets called modules or communities, whereby the nodes within modules are more highly connected with each other than with nodes outside the module. We used the Louvain algorithm, an agglomerative method, to detect the optimal number of modules within the network [42]. The maximum *Q*-value was used to determine modularity, which is calculated as the number of intramodule connections compared to the intermodule connections that would be expected by chance in a random network with the same number of modules. We describe the module membership for each network at minimum density by examining the nodes that comprise each module and how the relative compositions may differ. The minimum density was selected because it reflects the most conservative network composition (e.g., retains only the strongest positive correlations needed to construct a fully connected graph, thereby minimizing inclusion of spurious correlations) for descriptive purposes. BrainNet Viewer [43] was used to visualize the networks for each cognitive cluster, with nodes plotted by module membership and degree.

### Brain network comparisons

To compare differences in the network properties constructed within each cognitive cluster, we used the analytic tools provided in the GAT application. One particular challenge in comparing network coefficients is that they are computed at a range of densities and there is no good rationale for choosing one particular density value over another. Moreover, if one were to statistically test differences in coefficients at each density, this would introduce the problem of multiple comparisons and potentially complicate data interpretation with divergence of results across density values. To overcome these challenges, we analyzed network differences in global topological measures by integrating data for a given coefficient across the full range of densities using a commonly implemented approach called Functional Data Analysis [44,45]. FDA is similar to an Area Under the Curve analysis whereby the coefficients plotted across the range of densities create a curve that is represented by a function, and the area between the curves for each group can be statistically compared using a non-parametric permutation test. Permutations were conducted by randomly reassigning each of the 148 brain regions (i.e., residuals for cortical thickness or gyrification) to one of three new groups, while preserving the original sample sizes for each group, and this was repeated 1000 times. For each permutation, binary adjacency matrices were generated and thresholded across the aforementioned range of densities and network coefficients were calculated at each density. The FDA curve functions were generated and the difference between the curves for all possible comparisons (Clusters: 1 vs. 2; 1 vs. 3; 2 vs. 3) was computed and used to construct null distributions. FDA values derived from the current network analysis were compared against the corresponding null distribution, generating *p*-values based on percentile position. The same FDA approach was conducted for comparing groups on regional network measures (one for each of the 148 ROIs), but given the large number of comparisons, an additional False Discovery Rate correction (*p* < .05) was applied. Hubs were identified as nodes whose FDA-based node degree or node betweeness was two or more standard deviations above the mean FDA-based node degree or betweeness.

## Results

### Cognitive cluster analysis

**Fig 1:**
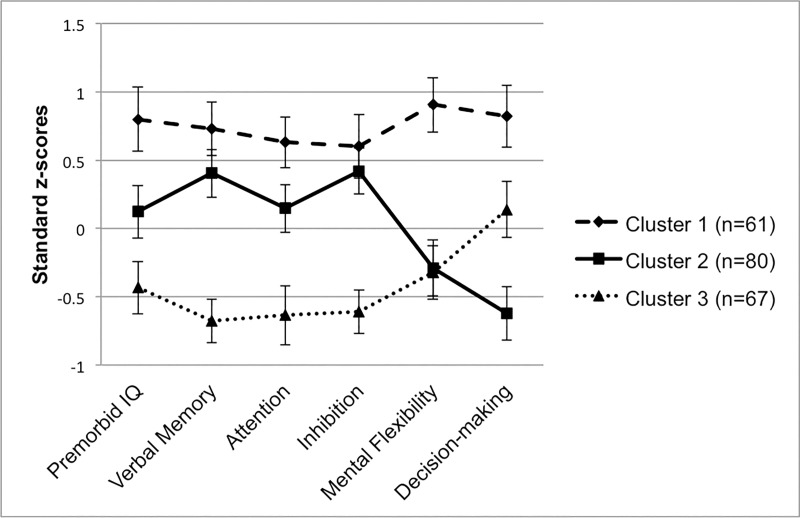
Neurocognitive profiles. Clusters plotted by mean neurocognitive score in standard z-score units based on sample mean and standard deviation. Error bars represent 95% confidence intervals.

**Fig 2:**
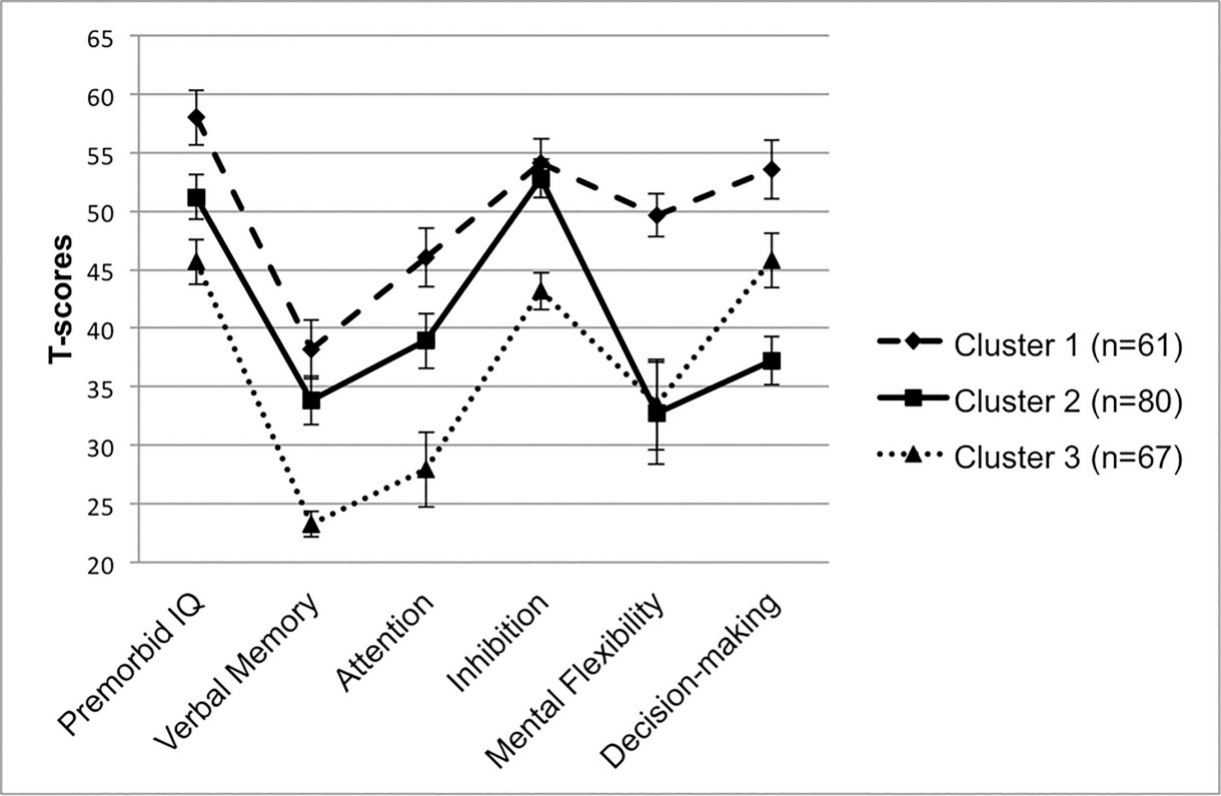
Demographic-adjusted neurocognitive profiles. Clusters plotted by mean neurocognitive score in T-score units corrected for age and/or education using normative test databases. Error bars represent 95% confidence intervals.

The three cognitive groups generated from the cluster analysis of 299 participants were reconstructed with the reduced sample (*n* = 208) included in the brain network analysis and depicted in Fig 1. Cluster 1 (*n* = 61) was the smallest group, but demonstrated relatively higher cognitive functioning relative to the other groups. Cluster 2 (*n* = 80) fell closest to the sample mean across many domains, but exhibited a prominent weakness in executive functions. Cluster 3 (*n* = 67) demonstrated overall poorer functioning relative to the others, with the exception of a relative strength in decision-making. To provide a benchmark for level of clinical impairment, the groups were plotted by mean T-scores corrected for age (and education where available for a given test) based on the normative databases for each test (see Fig 2). Sample characteristics of each cluster are outlined and compared in Table 1.

**Table 1:** Neurocognitive cluster characteristics.

	Cluster 1 (*n* = 61)	Cluster 2 (*n* = 80)	Cluster 3 (*n* = 67)	Cluster Comparisons
Age years, *M* (SD)	43.5 (9.4)	42.9 (9.7)	41.4 (9.8)	ns
Education years, *M* (SD)	10.9 (2.3)	10.2 (2.5)	10.1 (1.9)	ns
Monthly income (CAD), *M* (SD)	845.8 (475.7)	858.7 (454.0)	830.0 (322.6)	ns
Duration on DTES (years), *M* (SD)	6.7 (5.2)	8.0 (7.1)	8.9 (7.3)	ns
Male sex, % (*n*)	91.8 (56)	68.8 (55)	86.6 (58)	C2 < C1, C3***
Ethnicity, % (*n*)				
White	80.3 (49)	61.3 (49)	47.8 (32)	C1 > C2, C3***
Indigenous	13.1 (8)	27.5 (22)	38.8 (26)	C1 < C2, C3**
Other	6.6 (4)	11.3 (9)	13.4 (9)	ns
Psychotic Disorder, % (*n*)				
Schizophrenia spectrum	6.6 (4)	21.3 (17)	20.9 (14)	C1 < C2, C3*
Substance induced	9.8 (6)	12.5 (10)	20.9 (14)	ns
Other psychosis	18.0 (11)	8.8 (7)	14.9 (10)	ns
Charlson Comorbidity Index, *M* (SD)	3.2 (2.8)	3.2 (3.0)	3.4 (3.1)	ns
Substance Dependence, % (*n*)				
Alcohol	14.8 (9)	12.5 (10)	22.4 (15)	ns
Cannabis	34.4 (21)	37.5 (30)	37.3 (25)	ns
Stimulant	85.2 (52)	88.8 (71)	79.1 (53)	ns
Opioid	41.0 (25)	48.8 (39)	31.3 (21)	ns
Viral infection^a^				
HIV	8.3 (5)	13.8 (11)	20.9 (14)	ns
Hepatitis C (antibody positive)	73.3 (44)	63.8 (51)	65.2 (43)	ns
Self-reported TBI, % (*n*)	72.1 (44)	61.3 (49)	58.2 (39)	ns

*Note*. ns = not significant; CAD = Canadian Dollars; DTES = Downtown Eastside; TBI = traumatic brain injury. Other psychosis = Psychosis not otherwise specified, Major Depressive Disorder with psychosis, or Bipolar Disorder with psychosis.

^a^n = 60 in Cluster 1.

*p < .05

**p < .01

***p < .005

### Cortical thickness network analysis

**Fig 3:**
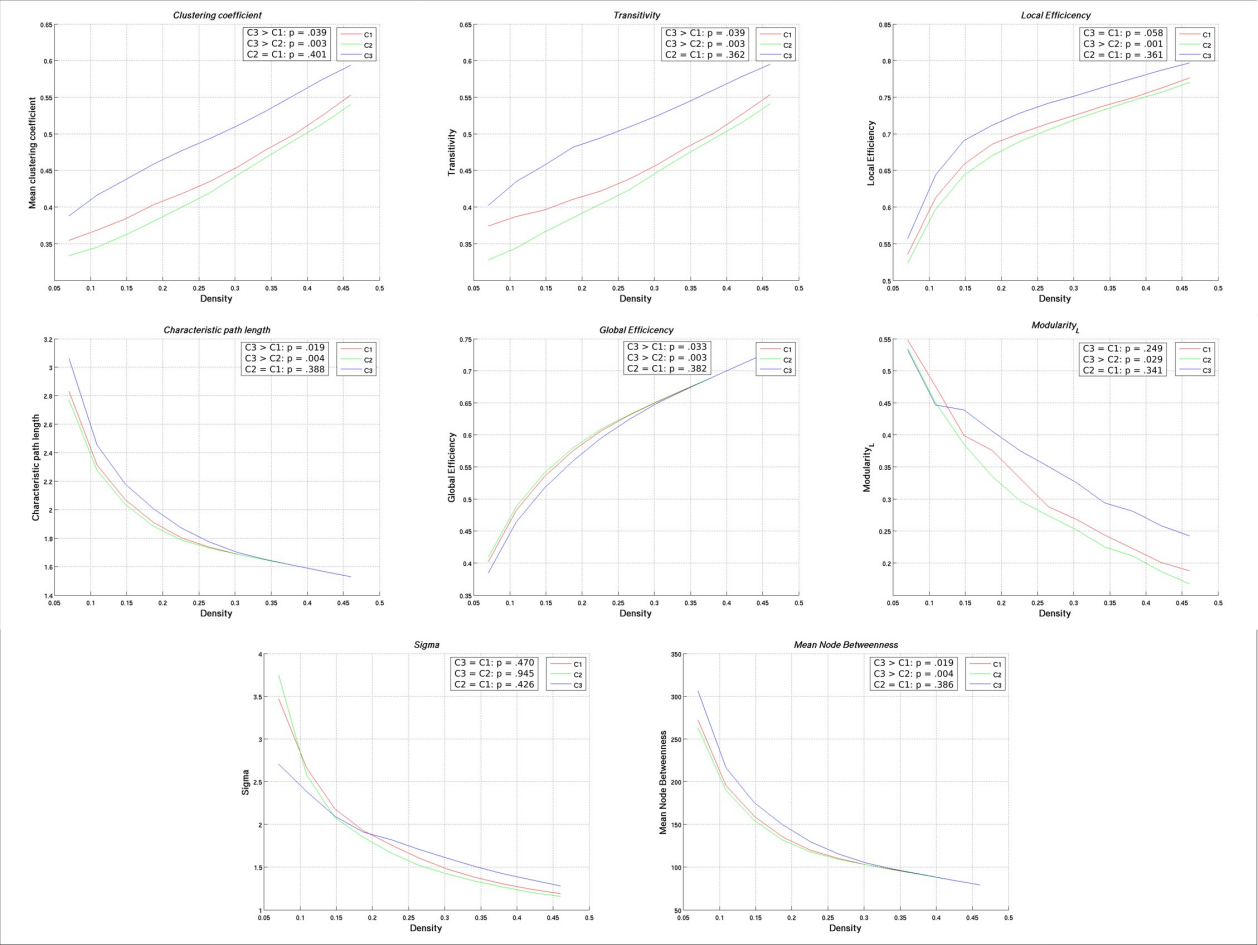
Functional data analysis curves for cortical thickness networks. Mean cortical thickness network coefficients for neurocognitive clusters plotted as a function of density. Cluster comparisons and p-values are derived from results of the Functional Data Analysis. Sigma = small-world.

Participants who were included (n = 208) versus excluded (n = 142) from the network analysis only differed on mean estimated premorbid FSIQ (Included: *M* = 98.2, *SD* = 8.6; Excluded: *M* = 95.1, *SD* = 8.7). When the cognitive groups were compared on global network measures, Cluster 3 consistently differed from Clusters 1 and 2. Regarding measures of network segregation, Cluster 3 demonstrated a significantly higher clustering coefficient and transitivity compared to Clusters 1 and 2, whereas significantly higher local efficiency and modularity were observed only when compared to Cluster 2. For measures of integration, Cluster 3 exhibited a significantly longer characteristic path length and lower global efficiency compared to Clusters 1 and 2. No significant group differences were observed for small-worldness, and all networks were considered to have small world architecture. No differences were observed between Clusters 1 and 2 on any global network measure. Results of network comparisons are summarized in Table 2. Mean network coefficients plotted as a function of density for each subgroup are provided in Fig 3.

**Table 2:** Cortical thickness network comparisons of topological measures.

	Cluster 1	Cluster 2	Cluster 3	Significant FDA Comparisons (*p*-value)
Mean Global Coefficients				
Clustering coefficient	0.443	0.427	0.494	C3>C1 (.039); C3>C2 (.003)
Transitivity	0.450	0.429	0.507	C3>C1 (.039); C3>C2 (.003)
Local efficiency	0.696	0.687	0.723	C3>C2 (.001)
Modularity	0.322	0.291	0.359	C3>C2 (.029)
Characteristic path length	1.882	1.866	1.945	C3>C1 (.019); C3>C2 (.004)
Global efficiency	0.608	0.610	0.600	C3<C1 (.033); C3<C2 (.003)
Small-world	1.835	1.801	1.798	—
Mean Regional Coefficients				
Node betweeness	131.495	129.108	140.900	C3>C1 (.019); C3>C2 (.004)

*N* = 208. Coefficients for each cluster represent the mean network value averaged across densities. FDA = Functional Data Analysis; C1 = Cluster 1; C2 = Cluster 2; C3 = Cluster 3.

Comparison of regional network measures revealed higher node betweeness for select regions in Cluster 3 when compared to Clusters 1 and 2 (see Table 3). No differences were observed for node degree or when comparing Cluster 1 versus 2 at the FDR-corrected level. Hubs identified for each network on the basis of node betweeness are listed in Table 4. Cluster 1 was characterized by four hubs distributed across the right hemisphere in occipital, temporal, and frontal regions. Cluster 2 also showed four hubs but these were located exclusively within the frontal region, bilaterally. In contrast, Cluster 3 had nearly twice as many hubs (i.e., seven) located primarily within occipital and temporal regions of the left and right hemispheres.

**Table 3:** Cortical thickness network nodes with altered betweeness.

Nodes	Significant Comparisons (*p*-value)
LH central sulcus	C3 > C1 (.049); C3 > C2 (.037)
LH lingual gyrus	C3 > C1 (.049)
LH inferior temporal gyrus	C3 > C2 (.037)
LH posterior segment of the lateral sulcus	C3 > C2 (.037)
RH inferior frontal sulcus	C3 > C2 (.037)
RH medial orbital sulcus	C3 > C1 (.049)

*Note*. FDR-corrected *p*-values. LH = left hemisphere; RH = right hemisphere.

**Table 4:** Cortical thickness network hubs defined by node betweeness.

Cluster 1	Cluster 2	Cluster 3
RH calcarine sulcus	RH inferior frontal sulcus	LH inferior temporal sulcus
RH circular sulcus of the insula	RH vertical ramus of the lateral sulcus	LH posterior segment of the lateral sulcus
RH medial orbital sulcus	RH H-shaped orbital sulcus	LH circular sulcus of the insula
RH suborbital sulcus	LH transverse frontopolar sulcus and gyrus	LH superior temporal sulcus
		RH posterior dorsal cingulate gyrus
		RH circular sulcus of the insula
		RH anterior transverse collateral sulci

*Note*. Hubs defined by node betweeness ≥ 2 standard deviations above mean cluster network node betweeness. RH = right hemisphere; LH = left hemisphere.

**Fig 4:**
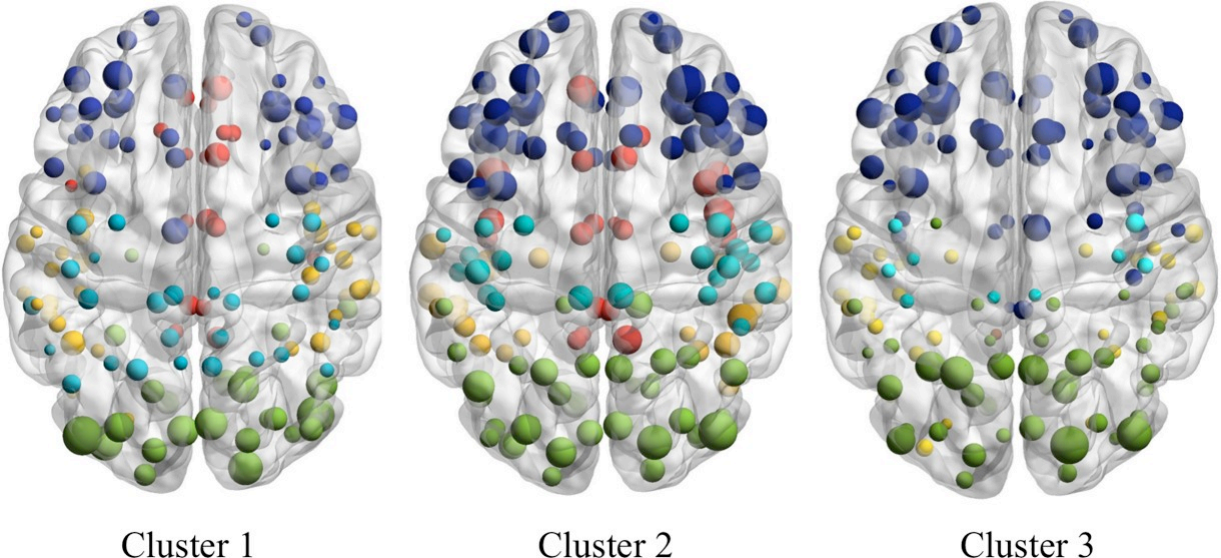
Cortical thickness network visualization. Visualization of cortical thickness networks for each neurocognitive cluster. Nodes are plotted by module membership. Greater node size reflects higher node degree (i.e., a greater number of connections). Module 1 (limbic lobe) = red; Module 2 (temporo-occipital) = yellow; Module 3 (occipital) = green; Module 4 (parieto-frontal) = light blue; Module 5 (frontal) = dark blue.

Five modules were identified for each network at the minimum density. The relative distribution of nodes across the modules, along with relative node degree, are visualized in Fig 4. Upon closer inspection, Module 1 was mostly characterized by nodes located within the limbic lobe. This module was very sparsely populated in Cluster 3, with only two nodes. The majority of the limbic lobe nodes that were interconnected in Clusters 1 and 2 were instead assigned to a frontal module in Cluster 3. Module 2 was predominately composed of occipito-temporal nodes in all three networks, with evidence of additional connectivity extending to the insula region in Cluster 1. Module 3 was comprised of nodes largely located in the occipital lobe within each network, but there appeared to be a few distributed connections to frontal and temporal regions for Cluster 2 and Cluster 3. Module 4 was represented by nodes that were more widely distributed across parietal and lateral frontal regions, but again this module was sparsely populated in Cluster 3 compared to the other groups. Lastly, Module 5 was characterized as predominately frontal with some connectivity to insula and limbic regions, and this module was more densely populated in Cluster 3 compared to the other groups.

### Gyrification network analysis

**Fig 5:**
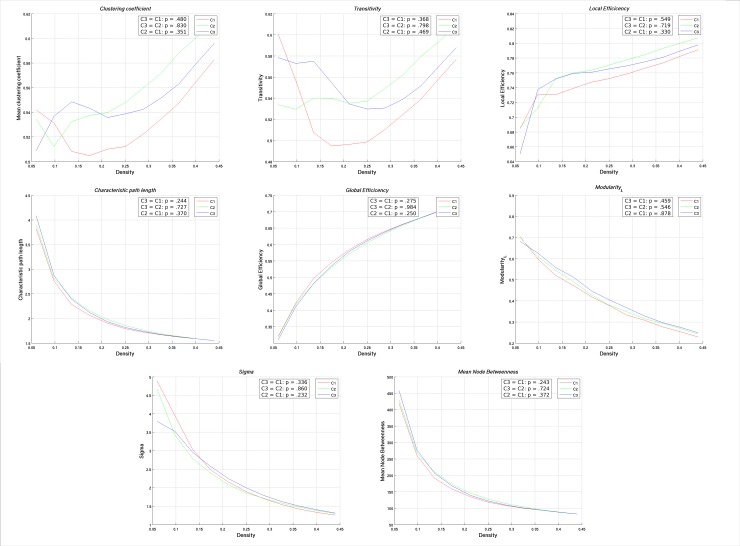
Functional data analysis curves for gyrification networks. Mean gyrification network coefficients for neurocognitive clusters plotted as a function of density. Cluster comparisons and p-values are derived from results of the Functional Data Analysis. Sigma = small-world.

**Fig 6:**
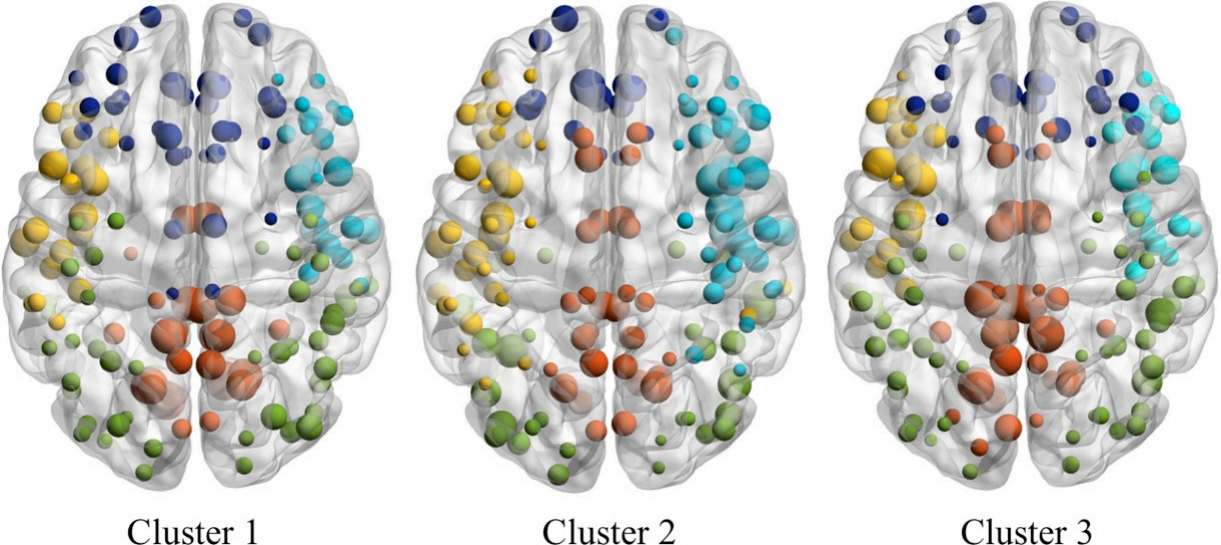
Gyrification network visualization. Visualization of gyrification networks for each neurocognitive cluster. Nodes are plotted by module membership. Greater node size reflects higher node degree (i.e., a greater number of connections). Module 1 (occipito-parietal/midline) = red; Module 2 (fronto-temporal, left hemisphere only) = yellow; Module 3 (occipito-parietal/limbic) = green; Module 4 (fronto-temporal, right hemisphere only) = light blue; Module 5 (frontal) = dark blue.

Across the three cognitive groups, no differences were found between any global or regional network measures of gyrification. For descriptive purposes, mean network coefficients are summarized in Table 5 and plotted as a function of density for each group in Fig 5. All networks demonstrated small world architecture. A relatively equal number of hubs based on node betweeness were identified for each group, which is outlined in Table 6. Similar to the cortical thickness networks, five modules were identified at the minimum density for each cluster. The distribution of nodes across the modules and relative node degree is depicted in Fig 6. Visual comparison of these figures suggests that the nodular composition of each module is relatively similar across networks. Module 1 consisted of nodes located predominately within midline structures spanning from occipital to parietal and frontal regions. Modules 2 and 4 were comprised of fronto-temporal nodes, but exclusively within the left and right hemispheres, respectively. Module 3 was predominately occipital and parietal nodes but with some extension to the limbic region. Lastly, Module 5 was considered to be predominately frontal.

**Table 5:** Gyrification network comparisons of topological measures.

	Cluster 1	Cluster 2	Cluster 3	Significant FDA Comparisons (*p*-value)
Mean Global Coefficients				
Clustering coefficient	0.5328	0.5581	0.5495	ns
Transitivity	0.5330	0.5553	0.5567	ns
Local efficiency	0.7506	0.7643	0.7580	ns
Modularity	0.4038	0.4030	0.4252	ns
Characteristic path length	2.0709	2.1213	2.1267	ns
Global efficiency	0.5809	0.5744	0.5755	ns
Small-world	2.3316	2.2418	2.2466	ns
Mean Regional Coefficients				
Node betweeness	159.4865	166.9582	167.7482	ns

*N* = 208. Coefficients for each cluster represent the mean network value averaged across densities. All cluster comparisons had p-values > .232. FDA = Functional Data Analysis; ns = not significant.

**Table 6:** Gyrification network hubs defined by node betweeness.

Cluster 1	Cluster 2	Cluster 3
LH planum polare	LH superior suborbital sulcus	LH middle anterior cingulate sulcus and gyrus
LH lateral orbital sulcus	LH superior frontal gyrus	RH triangular part of the inferior frontal gyrus
RH superior occipital gyrus	RH fronto-marginal sulcus and gyrus	RH temporal pole
RH occipital pole	RH orbital gyri	RH H-shaped orbital sulcus
RH anterior circular sulcus of the insula	RH occipital pole	RH occipital pole
RH posterior transverse collateral sulci	RH medial occipital-temporal sulcus & lingual sulcus	RH medial occipital-temporal sulcus & lingual sulcus
	RH H-shaped orbital sulcus	

*Note*. Hubs defined by node betweeness ≥ 2 standard deviations above mean cluster network node betweeness. RH = right hemisphere; LH = left hemisphere.

## Discussion

We identified three distinct profiles of cognitive functioning that were associated with underlying differences in cortical brain network topology in a large sample of homeless and vulnerably housed adults with significant multimorbidity. Using structural covariance network analyses of complementary cortical parameters, we found significant between-group differences in network coefficients for cortical thickness. Specifically, we found that Cluster 3 (the lowest cognitive functioning group) was significantly higher on measures of segregation (clustering coefficient, transitivity, local efficiency, modularity) and demonstrated reduced network integration (longer characteristic path length, lower global efficiency) compared to the other groups for the cortical thickness networks. All networks demonstrated a small-world architecture. Regionally, Cluster 3 exhibited higher node betweeness and had a greater number of hubs overall compared to Clusters 1 and 2. No differences were observed between Clusters 1 and 2 in any comparison. At minimum density, five modules were identified for each cortical thickness network, with noticeably different patterns of node distribution across modules within each group.

In comparison, no between-cluster differences were found on any gyrification network coefficients. The number of hubs identified in each cluster was relatively even, and tended to be greater in number compared to hubs identified in cortical thickness networks (for Clusters 1 and 2). Five modules were also identified for gyrification networks, but the nodal composition of modules within each cluster appeared highly consistent and more spatially compact compared to cortical thickness networks. These overall differences in network structures suggest that cortical thickness, but not gyrification, may be a sensitive marker of cognitive dysfunction, and associated multimorbid illness, in a marginalized population.

Convergent findings across different cortical thickness network measures suggest that the poorer profile of cognitive functioning in Cluster 3 may reflect an underlying disruption of the balance between network segregation and integration, in keeping with literature showing associations between network efficiency and intellectual functioning [12,13,16]. Segregation reflects a tendency towards functional specialization within a complex system, but this must be optimally balanced with integration of information between ostensibly discrete units (i.e., modules), an equilibrium that ultimately gives rise to the small-world property. One context in which increased segregation is seen is in early development, whereby the maturational trajectory of structural and functional networks of healthy humans follows a pattern of decreasing segregation in favor of increasing global integration, while preserving a small-world architecture [46,47]. Decreasing interregional correlation (i.e., segregation) has been shown to be most pronounced in association cortex during adolescence, which may reflect the dynamic pruning processes and myelination that occurs during this developmentally sensitive period [48]. In disorders of disrupted neurodevelopment, there are reports of network alterations in patients with schizophrenia characterized by increased clustering and longer path lengths in cortical thickness networks [49] and increased regional clustering in the dorsolateral prefrontal cortex in gyrification networks [39]. Other studies have reported decreased integration, but not increased segregation in patients with schizophrenia [50], with evidence for preserved small-worldness [51].

We also observed higher centrality (mean node betweeness) in Cluster 3 compared to Clusters 1 and 2, which may reflect a compensatory mechanism for reduced global integration. Higher betweeness centrality is thought to index influential nodes within a network that act as connectors between disparate parts of the system [40]. Regionally, Cluster 3 demonstrated nearly twice as many hubs (as defined by node betweeness) when compared to the other groups, and further exhibited higher betweeness in nodes distributed across frontal, temporal, and occipital regions. It is possible this property may emerge as a function of increased average path length in order to maintain sufficient network-wide parallel processing of information. Higher node betweeness has been previously reported in primary and paralimbic cortices; and in frontal, temporal, and parietal regions of persons with schizophrenia compared to healthy controls [49,52], reflecting possible regional markers of network inefficiency.

Examination of the modular composition may further elucidate the observed patterns of altered network topology in Cluster 3. Modularity is considered the “hallmark of complex systems”, facilitating robustness, adaptability, and functional specialization [53]. Descriptively, the nodes that comprised each of the five modules in Cluster 1, and to a lesser extent Cluster 2, were more spatially proximal to each other than those observed for Cluster 3. For example, Module 1 was primarily composed of nodes located within the limbic lobe for Clusters 1 and 2, but Module 1 was defined by only two nodes in this region for Cluster 3. Instead, we observed that the limbic nodes in Cluster 3 were grouped within Module 5, which was composed of predominately frontal lobe nodes, suggesting there is greater connectivity of nodes in Cluster 3 that are spatially distal to each other and therefore incur greater material and metabolic connectivity costs [53]. The overall pattern of less spatial compactness in the frontal lobe of Cluster 3 could be taken as a signature of the poor executive functioning that characterizes this cognitive group.

It is interesting to consider whether the previous findings of Cluster 3 exhibiting higher rates of neurodevelopmental markers (i.e., schizophrenia diagnosis, neurological soft signs) [8] may be manifest in the current cortical thickness network architecture. Although we previously reported localized increases in fronto-temporal gyrification of Cluster 3 compared to Clusters 1 and 2 that were taken as putative markers of aberrant neurodevelopment [8], we did not find group differences in gyral covariance patterns in the current study. This may suggest that localized abnormalities in gyrification are subtle and do not have direct bearing on global network architecture, consistent with findings reported by Palaniyappan and colleagues [39].

Likewise, certain individual risk factors may not directly influence global network architecture. Despite previously documented differences between Clusters 1 and 2 with respect to sex, years of education, monthly alcohol use, and MRI pathology (stroke, aneurysm) [6,8], cortical thickness and gyral covariance patterns were highly similar. Neurodevelopmental aberrations and/or higher overall illness burden (i.e., multimorbidity) may be clinical features that have a more pervasive impact on structural networks, as seen in relation to Cluster 3. Specifically, changes in structural covariance patterns may be more reflected in cortical thickness, a parameter that is highly sensitive to the cumulative lifetime effect of environmental risk exposures such as substance use [54], HIV infection [55] and traumatic brain injury [56], and progression of psychiatric illness [57]. The efficiency of networks derived from cortical thickness has been shown to be reduced in adults experiencing greater environmental deprivation [58]. Cortical thickness networks, but not gyral networks, may therefore be an important indicator of the neural consequences of multimorbidity in the context of marginalization and warrants future examination within a network science framework.

One important limitation of the current study is that a biologically meaningful interpretation of structural covariance networks is challenging. Brain network science is an emerging and rapidly evolving field, but an understanding of what the network coefficients can reliably reveal about the neurobiological underpinnings at multiple different systems levels (e.g., molecular, cellular) is incomplete. Accumulating work suggests that covariance of brain regions can be used to infer some aspects of connectivity [11], with approximately 35–40% of positive cortical thickness correlations overlapping with defined white matter tracts in the human brain [36]. Another important caveat is that we were not able to statistically compare the node composition of each module across the cognitive groups, but rather provided a description of the relative differences of node distribution. Any interpretation of a possible link between modularity and cognitive functioning in this study must be made with caution.

Relatedly, because we used a group-level analysis, we were precluded from being able to directly associate network parameters with individual measures of cognitive functioning or clinical variables. An individualized structural network approach would be complementary. Lastly, although our population of focus has shared features with other marginalized populations, most notably extreme morbidity and mortality [1], there are also likely unique features of our sample that reflect influences of the local environment and this may somewhat limit the generalizability of our findings. For example, participants are recruited from a single neighbourhood with a high concentration of subsidized housing in significant disrepair [3], and a high degree of economic disparity such that the neighbourhood has been referred to as the “poorest postal code” in Canada [59]. Nonetheless, we report similar sociodemographic, clinical, and cognitive characteristics as other Canadian studies of homeless and vulnerably housed adults [7,19,20], but substance use policies and access to healthcare vary outside of the Canadian context.

The current work represents a novel and important advancement in understanding the neuropsychological status of homeless and marginalized persons by identifying differential network topologies of well-defined cognitive groups using complementary cortical parameters. The overall pattern of results is consistent with the very eloquently expressed idea that “Impairment or loss of cognitive functions with disease can be accounted for by abnormal trade-offs that have an impact on often preferentially the most costly components of the networks that are also the most important for integrative processing and adaptive behaviour” (p. 347) [60]. Homeless and unstably housed persons face numerous risk factors for brain and cognitive dysfunction, and network science offers a valuable systems-based approach to understanding brain-behaviour relationships in a clinically heterogeneous population. Our findings suggest that covariance patterns of cortical thickness may represent important structural phenotypes that requires further examination. Importantly, networks are flexible and can be reconfigured with new learning [61]. Future work in this area should continue to focus on establishing structural networks as valid markers of cognitive functioning, as well as identifying the genetic and environmental risk factors that modify networks and rehabilitative interventions that can optimally rewire networks.
